# AAVs targeting human carbonic anhydrase IV enhance gene delivery to the brain

**DOI:** 10.1016/j.celrep.2025.116419

**Published:** 2025-10-31

**Authors:** Changfan Lin, Xinhong Chen, Jonathan D. Hoang, Fiona Ristic, Yujie Fan, Seongmin Jang, Jin Hyung Alex Chung, Erin E. Sullivan, Tomasz Gawda, Bill Kavvathas, Irene Tran, Yitong Li, Andrew D. Steele, Timothy F. Shay, Viviana Gradinaru

**Affiliations:** 1 Division of Biology and Biological Engineering, California Institute of Technology, Pasadena, CA, USA; 2 Department of Biological Sciences, California State Polytechnic University Pomona, Pomona, CA, USA

## Abstract

Gene therapies using natural adeno-associated virus (AAV) serotypes have restricted applications, particularly in the brain, due to their poor targeting and resulting safety concerns. Directed evolution has identified brain-enhanced engineered AAV capsids for various model organisms, but inter-species differences challenge their translation. Here, we engineer AAVs to target human carbonic anhydrase IV (CA-IV), a recently identified blood-brain barrier transcytosis receptor with several favorable properties. We perform *in vitro* on-column selection of an AAV library to exclude capsids that do not target human CA-IV. We subsequently screen the top 0.01% of variants *in vivo* in mice expressing human CA-IV in brain endothelial cells. Notably, two top-performing capsids in “humanized” mice are relatively lower ranked *in vitro* and outperform several top-ranked *in vitro* candidates. One of these, AAV-hCA4-IV77, achieves 100-fold-greater brain transduction than AAV9, with robust neuronal and astrocytic coverage across brain regions. These results advance our understanding of receptor-targeted capsid design and support the therapeutic potential of human CA-IV-engaging AAVs.

## In brief

Adeno-associated viral vectors (AAVs) based on natural serotypes poorly transduce the brain after intravenous administration, limiting clinical efficacy. Lin et al. engineer AAV9 to engage the human version of CA-IV, a blood-brain barrier receptor used previously by potent but mouse-specific vectors, and validate their function in a “humanized” mouse model.

## Graphical Abstract

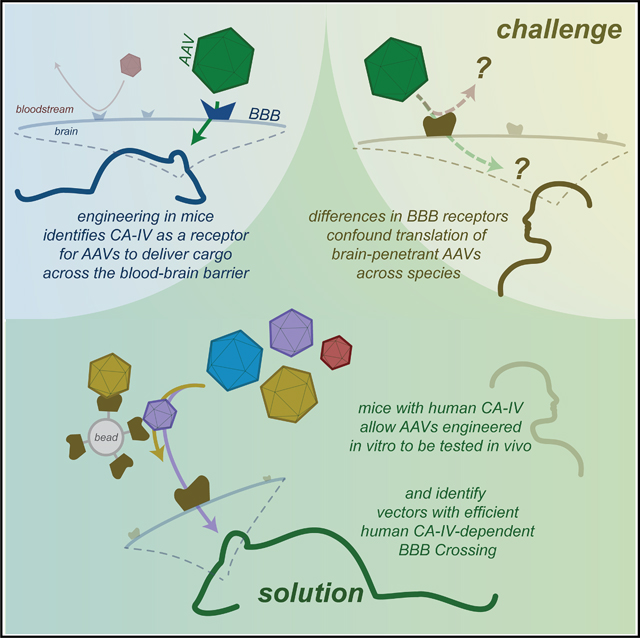

## INTRODUCTION

ene therapy is a rapidly advancing field that treats genetic disorders by addressing their molecular origins. Adeno-associated viral vectors (AAV) are preferred gene delivery vehicles due to their favorable safety profile compared to other viral vectors and their lack of known pathogenicity in humans.^1–3^ Currently, there are over 300 clinical trials exploring AAV-based therapies, and five treatments based on naturally evolved AAV serotypes have already received approval from the US Food and Drug Administration (FDA).^4–9^ These include Zolgensma, which utilizes the AAV9 capsid for intravenous targeting of the nervous system to treat spinal muscular atrophy in children up to 2 years of age.^10,11^ While gene therapies utilizing natural AAV serotypes are dramatically changing the trajectory of select genetic disorders, many cell types and tissues are transduced only at high systemic doses, negatively impacting the safety and efficacy of existing treatments and precluding application to some indications.^12–15^ Thus, for AAV-based gene therapy to reach its full potential, further optimized AAV vectors are required.

Recent advances in engineering AAV capsids for systemic delivery through *in vivo* directed evolution have markedly enhanced specificity and potency in mice and non-human primates (NHPs).^16–23^ This engineering approach involves introducing a diversified library of AAV capsid variants into an animal and recovering those that effectively reach the target tissues or cell types. The resulting engineered systemic AAVs have been broadly applied as research tools, which have revealed that the capsids’ properties are often specific to the model organism in which they were developed, with tropism changing or disappearing in different host genetic contexts.^24–26^ This limitation is primarily due to receptor differences between species. Performing directed evolution in NHPs increases the translational potential of engineered capsids, but it is resource-intensive and does not fully eliminate the risk of altered behavior when applied to humans. Thus, a different approach for engineering human-optimized systemic AAVs is needed.

**Figure 1.**
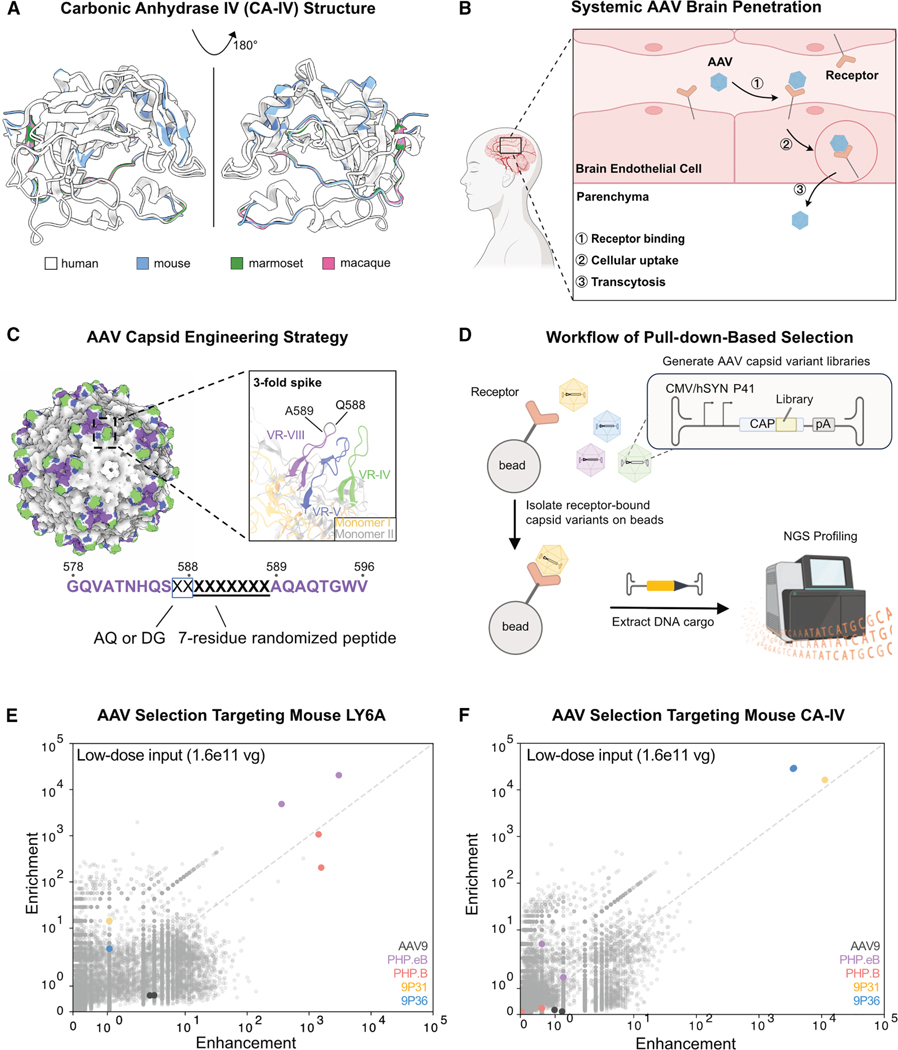
Human BBB receptor-targeted AAV capsid engineering pipeline (A) Structure of CA-IV from human (PDB: 1ZNC^39^), macaque (modeled by AlphaFold 3^40^), marmoset (modeled by AlphaFold 3), and mouse (PDB: 2ZNC^41^). Human CA-IV is shown in white. Regions that differ from human CA-IV in other species, and which may influence receptor binding by AAV variants, are highlighted in blue (mouse), green (marmoset), and magenta (macaque). (B) Illustration of critical stages of systemic AAV penetration into the brain from the bloodstream: (1) AAV binding to brain endothelial cell surface receptors; (2) cellular uptake of bound AAV through receptor-mediated internalization; and (3) transcytosis to enter the brain parenchyma. (C) Structure of the AAV9 capsid (PDB: 7MT0^41^), highlighting the 3-fold spike and key variable regions (VR) VR-IV (green, residues 445–465), VR-V (blue, residues 488–511), and VR-VIII (purple, residues 578–596). The enlarged panel shows the interaction of VR-IV and VR-VIII from the same AAV capsid monomer (colored yellow) with VR-V from a different monomer (colored light gray). To construct our library of AAV capsid variants, a seven-residue randomized peptide was inserted between positions 588 and 589 of VR-VIII, and positions 587 and 588 were modified to either AQ or DG. (D) Pull-down-based selection workflow used to identify AAV variants that bind target receptors. Capsid variants that bind to the receptor (e.g., CA-IV or LY6A) are isolated with receptor-bound beads, followed by DNA extraction and NGS to identify the enriched variants. (E and F) Validation of pull-down-based selection for AAV variants binding mouse LY6A (E) and CA-IV (F) receptors. AAVs known to bind LY6A (PHP.B and PHP. eB) and mouse CA-IV (9P31 and 9P36) show significantly higher Er and Eh than other AAVs in a pool of ∼18,000 unique variants. Identically colored dots represent codon replicates of an AAV variant.

The central nervous system (CNS) is an especially challenging target for gene therapy due to the blood-brain barrier (BBB). This protective structure, composed primarily of tightly joined endothelial cells, restricts the entry of most therapeutic agents into the brain.^27–31^ Recent efforts by our lab and others have identified the BBB receptors utilized by certain engineered systemic capsids to cross into the brain through receptor-mediated transcytosis.^32–36^ One such receptor is carbonic anhydrase IV (CA-IV), which facilitates the efficient BBB crossing of AAV variants 9P31 and 9P36 in mice.^18,32^ Importantly, CA-IV is also present in NHP and human BBB endothelial cells (Figures S1A and S1B)^37,38^ and exhibits a more CNS-focused expression pattern than other established BBB receptors, such as low-density lipoprotein receptor-related protein 6 (LRP6), transferrin receptor (TfR1), and insulin receptor (INSR) (Figure S1C). This restricted distribution may offer advantages for targeted drug delivery to the brain, potentially reducing off-target effects in other tissues. While CA-IV homologs exhibit significant sequence conservation across species, they are not identical. For instance, rhesus macaque and marmoset CA-IV share 85% amino acid identity with human CA-IV, whereas mouse CA-IV shares only 55% identity (Figure 1A). As a result, AAVs 9P31 and 9P36 bind exclusively to mouse, but not primate, CA-IV, with 9P31 crossing the BBB similarly to AAV9 in macaque.^42^

Here, we implemented an *in vitro* pipeline with on-column and in-cell selections, similar to that recently used for mouse LY6A^31^ and human TfR1,^43^ to identify human CA-IV-engaging AAVs. After narrowing down the pool of engineered AAVs *in vitro*, single capsid variants were characterized *in vivo* in a mouse model expressing human CA-IV in brain endothelial cells. We found that several top-ranked variants (Alpha 48, 49, and 62) have enhanced potency in human CA-IV-expressing cells yet poorly transduce the “humanized” mouse brain after intravenous administration, with potency akin to AAV9. Therefore, instead of a fully *in vitro* pipeline, we integrated *in vivo* selection with “humanized” mice following round 1 on-column selection. We identified two capsids among the top 0.01% of round 1 on-column performers (10,000 variants) that showed robust brain transduction in “humanized” mice despite ranking lower than Alpha 48, 49, and 62 in round 2 *in vitro* selections. These results inform future capsid engineering pipelines and support the translational potential of AAVs targeted to human CA-IV.

## RESULTS

### *In vitro* selections successfully identify known receptor-AAV pairs

The process of receptor-mediated BBB crossing by AAVs involves three key steps: receptor binding, cellular uptake, and transcytosis (Figure 1B).^31,44–46^ First, AAVs circulating in the bloodstream enter blood vessels in the brain and interact with their target receptors on endothelial cells. This interaction then mediates internalization of the AAVs into the endothelial cells. Finally, AAVs are transported through the cells and released into the brain parenchyma. While the mechanistic details of cellular uptake and transcytosis are poorly understood, mechanism-agnostic-directed evolution of AAV variants has led to the identification of several luminal brain endothelial receptors, such as LY6A, LRP6, and CA-IV, that enable efficient AAV delivery to the brain following intravenous injection in model organisms.^18,31–33,47^ Leveraging this understanding, we designed a receptor-directed *in vitro* engineering workflow to identify AAV capsid variants that effectively interact with validated BBB transcytosis receptors. Our approach builds upon recent successes targeting other BBB receptors (such as murine LY6A and widely expressed human TfR1).^31,43^

We first generated a diversified library of AAV variants by modifying the 3-fold spike of AAV9, a natural serotype widely recognized for its brain transduction after systemic administration (Figure 1C). The 3-fold spike is formed by three variable regions: VR-IV, VR-V, and VR-VIII. Applying well-established library design strategies,^17,18^ we inserted a randomized seven-amino-acid peptide between residues 588 and 589 in the VR-VIII region, combined with either AQ or DG at positions 587–588.^48^ As in our previously described M-CREATE method,^17^ a low-efficiency transfection of the resulting plasmids into AAV producer cells was performed to maximize the fraction of cells containing instructions for a single library variant and thereby minimizing the assembly of capsid variant chimeras.

We then used an on-column pull-down-based selection method to identify AAV variants within the library that target receptors (Figure 1D). We validated this approach using a library that included known receptor-binding AAVs spiked into a background of ∼18,000 unique AAV capsid variants identified through an unrelated prior selection to have AAV9-like HEK cell transduction in order to determine our ability to isolate true receptor-engaging capsids. Each AAV variant was included twice, with synonymous codon replicates of the same protein sequence, to assess the reproducibility of the subsequent results (sequences shown in Table S1). Mouse receptors LY6A and CA-IV were modified by replacing their C-terminal glycosylphosphatidylinositol (GPI) anchors with a hemagglutinin (HA) tag. We then incubated the AAV library pool with anti-HA magnetic resin containing each HA-tagged receptor. Following an overnight incubation (compared to the 3–4 weeks required for *in vivo* directed evolution), we washed and eluted the interacting capsid variants. To identify non-specific capsids, we performed the same protocol in parallel with anti-HA magnetic resin lacking any receptor.

We used next-generation sequencing (NGS) to evaluate the performance of AAV variants. To quantify the increase in each variant’s abundance post-selection, indicating interaction with the target receptor, we calculated its enrichment (Er, Equation 1):

(Equation 1) $$\text{Enrichment}\;(\text{Er})=\frac{A\text{i}(\text{post receptor selection}\;)}{A\text{i}(\text{input pool}\;)},$$

where Ai is the relative abundance of variant i, calculated as the percentage of variant i in the total pool.

To account for non-specific binding to the resin, we also evaluated each variant’s post-selection abundance with and without receptor by calculating its enhancement (Eh, Equation 2):

(Equation 2) $$\text{Enhancement}\;(\text{Eh}\;)=\frac{A\text{i (post receptor selection)}\;}{A\text{i (post selection without receptor)}\;}.$$

**Figure 2.**
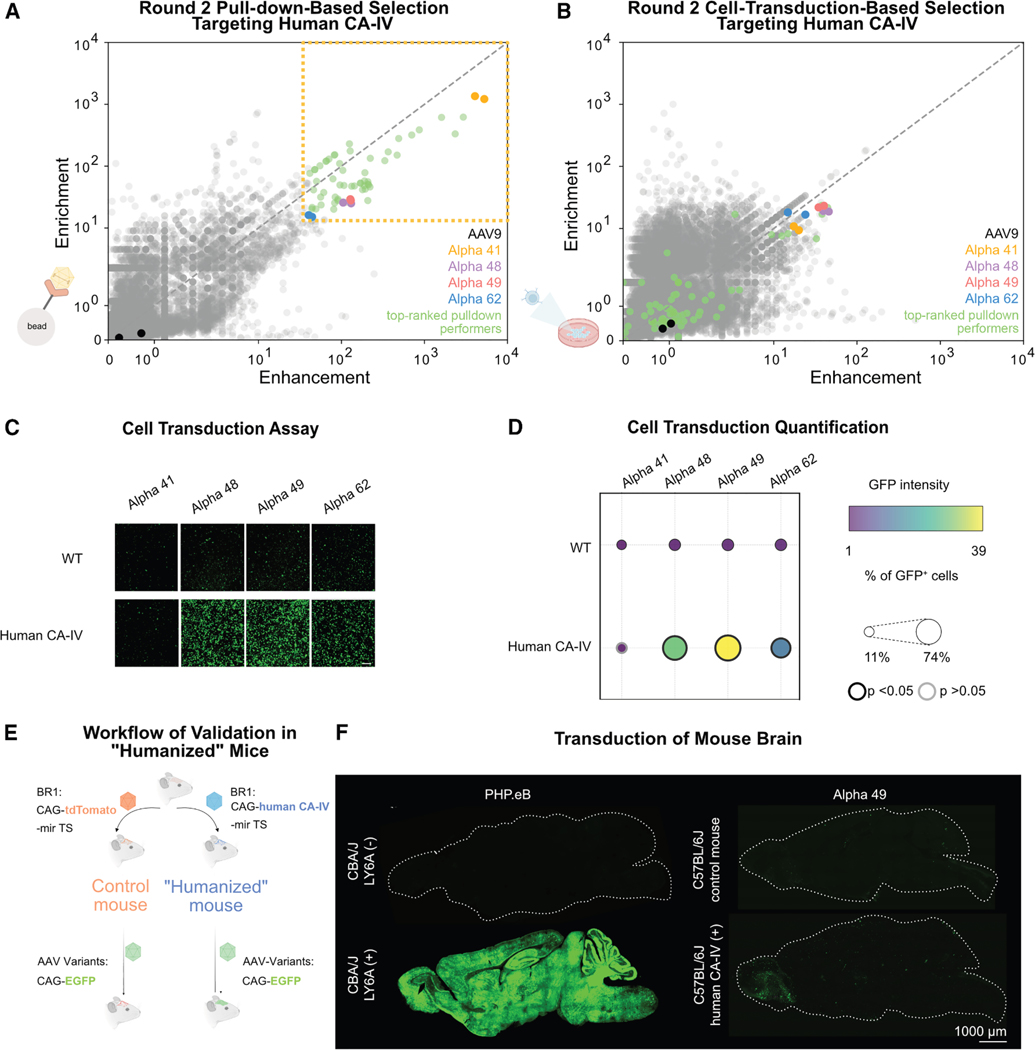
Identification of human CA-IV-targeting AAVs *in vitro* (A) Results of round 2 pull-down-based selection for AAV variants targeting human CA-IV. Promising candidates are highlighted, including the overall top-performing variants (Alpha 41, 48, 49, and 62), along with the control AAV9 for comparison. Top-performing AAVs are variants with both high Er and Eh scores (Er > 15 and Eh > 40; gold dotted box in top right). (B) Results of round 2 cell-transduction-based selection for AAV variants targeting human CA-IV. Promising candidates identified from round 2 pull-down-based selection (A) are highlighted, including the overall top-performing variants (Alpha 41, 48, 49, and 62), along with the control AAV9. (C) Transduction of wild-type (WT) and human CA-IV-expressing HEK293 cells by the overall top-performing AAV variants packaging EGFP. Fluorescence images show a significant increase in transduction with human CA-IV for all variants except Alpha 41. Scale bars, 100 μm. (D) Quantification of cell transduction (EGFP intensity and percentage of EGFP-positive cells), confirming enhanced transduction of Alpha 48, 49, and 62 in the presence of human CA-IV. An independent sample t test was performed between WT and human CA-IV expressing cells for each AAV variant. Dots with thick black borders represent a *p* value less than 0.05 in both EGFP intensity and percentage of EGFP-positive cells. Dots with gray borders did not meet this significance threshold. Dot size represents the percentage of EGFP-positive cells, with minimum and maximum values indicated in the legend. See Table S2 for quantification of transduction. (E) Workflow for validation of AAV variants in “humanized” mice. First, 1.5 × 10^12^ vg AAV-BR1 (BR1) carrying either CAG-human CA-IV or CAG-tdTomato (control) with microRNA-122 targeting sites (miR-TS, for reducing liver expression) was delivered to establish the expression of human CA-IV in brain endothelial cells. After 4 weeks, 5 × 10^11^ vg AAV variants carrying the EGFP transgene were administered, and their performance *in vivo* was evaluated after 3 weeks. (F) Representative images of brain sections comparing PHP.eB and Alpha 49 transduction in control mice versus mice expressing human CA-IV or LY6A. PHP.Eb demonstrates robust LY6A-dependent brain transduction, validating the model system for evaluating receptor-dependent AAV variants. Scale bars, 1,000 μm.

**Figure 3.**
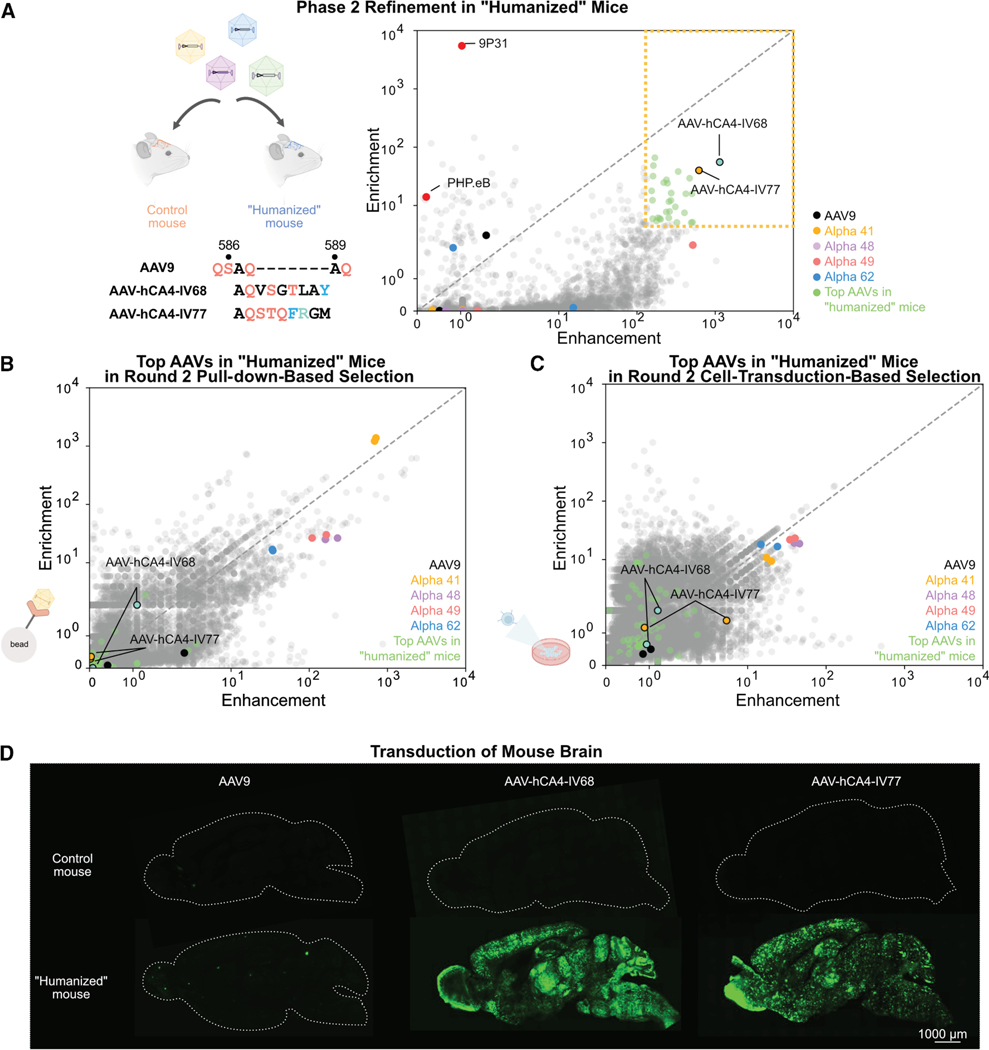
*In vivo* transcytosis-based selection to filter human CA-IV-engaging AAVs (A) Results of transcytosis-based selection in “humanized” mice expressing human CA-IV in brain endothelial cells. Identified variants AAV-hCA4-IV68 and AAV-hCA4-IV77 show high Eh and Er scores, while previously identified top *in vitro* performers (Alpha 41, 48, 49, and 62) demonstrate poor performance in this *in vivo* context. Control AAVs 9P31 and PHP.eB show high Er but low Eh scores, consistent with their documented brain transduction via mouse CA-IV and LY6A receptors, respectively. Top performing AAVs in “humanized” mice are variants with both high enrichment (Er) and enhancement (Eh) scores (Er > 5 and Eh > 150; colored light green). Amino acid sequences of AAV9 and the identified top-performing variants AAV-hCA4-IV68 and AAV-hCA4-IV77 are shown. The nine-residue modified regions (positions 586–589) are color-coded based on their properties: black, nonpolar aliphatic; blue, aromatic; red, polar uncharged; and green, positively charged. (B) Comparison of AAV performance in round 2 pull-down-based selection versus *in vivo* transcytosis selection. Scores for each variant were averaged across mice (*n* = 3). Notably, the top-performing variants in “humanized” mice (AAV-hCA4-IV68, AAV-hCA4-IV77, and light green colored variants) were not ranked highly in the pull-down selection. (C) Comparison of AAV performance in round 2 cell-transduction-based selection versus *in vivo* transcytosis selection. The top-performing variants in “humanized” mice (AAV-hCA4-IV68, AAV-hCA4-IV77, and light green colored variants) are highlighted. (D) Representative sagittal brain sections showing transduction by AAV9, AAV-hCA4-IV68, and AAV-hCA4-IV77 in control mice versus “humanized” mice expressing human CA-IV. To prevent saturation of the fluorescence signal in brain sections, images were captured using reduced illumination intensity. Both AAV-hCA4-IV68 and AAV-hCA4-IV77 demonstrate significantly enhanced and widespread transduction throughout the brains of “humanized” mice compared to control mice, while AAV9 shows consistently poor brain transduction in both groups. Scale bars, 1,000 μm.

Combined, these metrics provide an overall assessment of targeting efficiency and specificity that we term “performance” (performance thresholds for Er and Eh are indicated by gold dotted box in Figures 2A and 3A). We noticed one variant, Alpha 1 (nine-mer sequence AQYPPVFKS), that showed promising but inconsistent scores across codon replicates in a selection against mouse LY6A. The same variant appeared with low Eh scores consistent with non-specific binding but appreciable Er scores in a selection against mouse CA-IV (Figures S1D and S1E). It is known that LY6A is functional in C57BL/6J and DBA/2J strains of mice but not NOD/ShiLtJ,^34^ and that CA-IV is functional in all three strains.^32^ We therefore tested the tropism of Alpha 1 in these strains. Interestingly, Alpha 1 showed weakly enhanced endothelial tropism only in NOD/ShiLtJ mice (Figures S1F and S1G), inconsistent with the pattern of either receptor.

As expected, though, AAVs PHP.B and PHP.eB showed high Er and Eh values in selections with LY6A (Figure 1E). Their performance was consistent across both low (1.6 × 10^11^ viral genomes, vg) and high (8 × 10^11^ vg) doses of library input (Figure S1D). Similarly, mouse CA-IV effectively selected AAVs 9P31 and 9P36 (Figures 1F and S1E). The successful identification of known AAV-receptor interactions increased confidence in our selection method.

### *In vitro* identification of AAVs targeting human CA-IV

Having validated our method with known receptor-AAV pairs, we next turned to the identification of capsid variants that engage previously untargeted receptors, specifically human CA-IV. Following the same approach used above for LY6A, mouse CA-IV, and previously,^31,43^ we conducted one round of selection with our library of AAV9 variants with nine-residue modifications (randomized seven-amino-acid peptide insertion between residues 588 and 589 in the VR-VIII region,^17^ combined with either AQ or DG substitutions at positions 587–588). By selecting variants showing both Er and Eh scores greater than three, we efficiently narrowed the vast initial pool, previously estimated to be roughly 10^8^ variants (notably, undersampling the library’s theoretical chemical diversity),^17^ down to the top 0.01% (approximately 10,000) on-column performers.

To prioritize candidate AAV variants for individual validation, we first focused on the top-performing capsids during on-column selections with human CA-IV, as previous studies demonstrated that AAVs with high enrichment for receptors such as TfR1, LY6A, and LY6C1 exhibit efficient BBB crossing.^31,43^ Our experiments also confirmed this pattern, showing that the efficient mouse brain-transducing variant PHP.eB is strongly enriched and enhanced in LY6A pull-down selections, while 9P31 performs similarly for mouse CA-IV. To further evaluate the relative on-column performance of capsid variants, we performed a second round of pull-down-based selection. We produced a vector library of the top 10,000 candidates targeting CA-IV along with known AAVs (such as AAV9, PHP.eB, and 9P31) as controls, with each capsid variant represented by two synonymous codon replicates (Figure 2A). This selection identified a distinct group of top pull-down performers characterized by high enrichment (Er > 15) and enhancement (Eh > 40) scores (Figure 2A). The robustness of these results was confirmed by the strong correlation of Er and Eh scores observed between the codon replicates of these AAVs (Figure S2A).

Not all receptor-binding geometries may be compatible with cell internalization (Figure 1B, step 2). To further narrow down the list of on-column performers, we conducted an additional round of screening focused on cell transduction, using the same input pool (∼10,000 AAV variants) as for the round 2 pull-down selection. We transduced HEK293 cells transfected with either human CA-IV or a plasmid containing a scrambled sequence as a negative control and identified AAV variants with CA-IV-boosted transduction through RNA sequencing (Figure S2B). We calculated Er and Eh scores similarly to the pull-down selection, comparing variant abundance in human CA-IV-expressing cells to that in wild-type cells (Figure 2B). The separation between high- and low-performing variants was less than that in pull-down-based selections, underscoring the importance of a first round of pull-down-based selections for weeding out non-target-binding AAVs. Integrating our pull-down and cell transduction results yielded a refined list of four candidate variants for further testing: Alpha 41, 48, 49, and 62 (Figure S2C).

Individual characterization by surface plasmon resonance (SPR) with Fc-tagged human CA-IV attached to the chips revealed that all selected variants interact with human CA-IV (Figure S2D). While high avidity precludes accurate affinity determination, results were consistent with sub-nM apparent affinities. We also individually confirmed that identified variant capsids internalize into and transduce HEK293 cells in a human CA-IV-dependent manner. Indeed, human CA-IV-targeting AAVs Alpha 48, 49, and 62, but not Alpha 41, demonstrated CA-IV-enhanced transduction (Figures 2C and 2D; Table S2), although the degree of receptor-mediated enhancement was lower for Alpha 62 than for the other two capsid variants.

### A “humanized” mouse model shows that several top performers from on-column and in-cell selections fail to transduce the brain *in vivo*

Transcytosis is a necessary third step for BBB crossing (Figure 1B, step 3). Importantly, not all internalized AAVs complete this process. For example, AAV9-X1.1 targets LRP6 across species but primarily transduces endothelial cells rather than crossing the BBB in rodents, while in neonate NHPs, the same AAV binds the same receptor, resulting in effective transcytosis and neuronal targeting.^33,49^

To confirm transcytosis *in vivo*, we developed a “humanized” mouse model to investigate BBB crossing. We engineered C57BL/6J mice to express human CA-IV in their brain endothelial cells by systemically delivering either CAG-human CA-IV or CAG-tdTomato (negative control) using a brain endothelial-targeted AAV variant. To prevent potential liver toxicity,^50,51^ we incorporated a microRNA-122 target site (mir122-TS) into the cargo construct, which effectively reduces translation in hepatic tissue.^49,52^ After 4 weeks, we systemically administered our AAV candidates carrying the EGFP transgene and evaluated their *in vivo* performance 3 weeks later (Figure 2E).

To avoid the impact of neutralizing antibodies, which can significantly affect the efficacy of a second dose of AAV,^53^ during these sequential AAV administrations, we paired our AAV9-based capsids with the AAV2-based brain endothelial-targeted AAV-BR1.^53^ The evolutionary distance between these serotypes has previously been shown to enable efficient brain transduction after sequential administration.^53^ As a positive control, we intravenously administered 1.5 × 10^12^ vg of AAV-BR1 carrying either tdTomato (control) or LY6A to CBA mice, a strain known to lack the functional LY6A required for efficient PHP.eB brain transduction.^34,35^ Four weeks later, we similarly dosed the mice with 3 × 10^11^ vg of PHP.eB CAG-EGFP. As expected, after an additional 3 weeks, PHP.eB showed a strong dependency on LY6A for effective brain transduction. Only CBA mice engineered to express LY6A in their brain endothelial cells displayed robust EGFP expression across brain regions, while LY6A-deficient tdTomato-expressing controls exhibited minimal transduction (Figure 2F).

Following this validation of our model system, we evaluated the *in vivo* transcytosis efficiency of our engineered AAV variants Alpha 48, 49, and 62. We confirmed the expression of human CA-IV in brain endothelial cells, with distribution throughout the cortex, cerebellum, brainstem, thalamus, and hypothalamus (Figure S3A). Sparse neuronal transduction was also apparent in some brain regions, such as the cortex and thalamus. Surprisingly, however, we observed poor transduction of our top candidates from on-column and in-cell selections in both control and “humanized” mice expressing human CA-IV (Figures 2F and S3B).

### Round 2 *in vivo* selection identifies human CA-IV-targeting AAVs with efficient BBB transcytosis

This unexpected result prompted us to re-evaluate our engineering pipeline. In our fully *in vitro* selection process, practical constraints limited our ability to individually test all AAVs *in vivo*, leading us to prioritize top performing AAVs in human CA-IV pulldown and cell transduction selections (Alpha 48, 49, and 62). However, despite exhibiting enhanced transduction in cells expressing human CA-IV, these AAVs performed poorly *in vivo*.^54^

Therefore, rather than conducting a fully *in vitro* selection (round 1 on-column followed by round 2 parallel on-column and in-cell selections), we screened the top 0.01% of capsid performers from round 1 pull-down selection in a round 2 *in vivo* selection using our “humanized” mouse model (Figure 3A). We intravenously administered 3 × 10^11^ vg of these AAVs (∼10,000 variants), along with control AAVs (PHP.eB and 9P31). Each vector carried a neuronal synapsin promoter-driven transgene to ensure that when extracting RNA, we would selectively identify variants capable of crossing the BBB rather than those merely efficient at transducing brain endothelial cells (similar to the TRACER method^18^). We injected the AAV pool into both negative control mice expressing tdTomato and mice expressing human CA-IV (*n* = 3 per condition). Three weeks post-injection, we analyzed brain-enriched AAV transgene RNA using the same Er and Eh scoring metrics (Figure 3A).

The control AAVs 9P31 and PHP.eB showed high Er scores and low Eh scores, consistent with their documented robust brain transduction in mice via mouse CA-IV and LY6A receptors, respectively, and independent of human CA-IV. 9P31 demonstrated higher Er than PHP.eB, aligning with previous observations.^18^ Among the top 0.01% on-column performers, *in vivo* selection revealed two variants, AAV-hCA4-IV68 and AAV-hCA4-IV77 (hereafter abbreviated as IV68 and IV77), which outperformed several candidate vectors identified previously through fully *in vitro* selection (Alpha 48, 49, and 62) (Figure 3A). Interestingly, we noticed that these top-performing variants in the “humanized” mouse model (Eh score > 150 and Er score > 5) were not ranked highly within the top 0.01% of variants included in round 2 pull-down and cell transduction selections (Figures 3B and 3C). It is important to note that the Eh and Er scores serve as indicators rather than precise measurements of binding affinities.

When produced as single clones, IV77 exhibited about a 5-fold lower production yield compared to AAV9, and the addition of 300 mM NaCl to its buffer helped with stable storage, whereas AAV-hCA4-IV68 achieved yields comparable to AAV9 and required no such buffer formulation. Consistent with their cell transduction selection rankings, whereas Alpha variants showed strong human CA-IV dependent HEK cell transduction (Figure 2C; Table S2), IV68 and IV77 potencies were not affected by human CA-IV expression (Figures S4A and S4B; Table S3). Interestingly, despite the avidity of both the 60-mer capsid and dimeric receptor in the experimental design acting to boost apparent affinities, only IV77 could be confirmed to have binding to human CA-IV by SPR, with a maximum response (Rmax) roughly 30-fold lower than Alpha 49 (Figure S4C). As expected, IV77’s interaction is specific to human, not mouse, CA-IV. IV68’s strong *in vivo* performance despite its lack of SPR signal may stem from a weak/transient CA-IV interaction below the SPR detection threshold, suggesting that its interaction might be boosted *in vivo* via additional factors, e.g., additional receptors or CA-IV modifications.

### Identified AAV variants efficiently transduce the brain through human CA-IV

**Figure 4.**
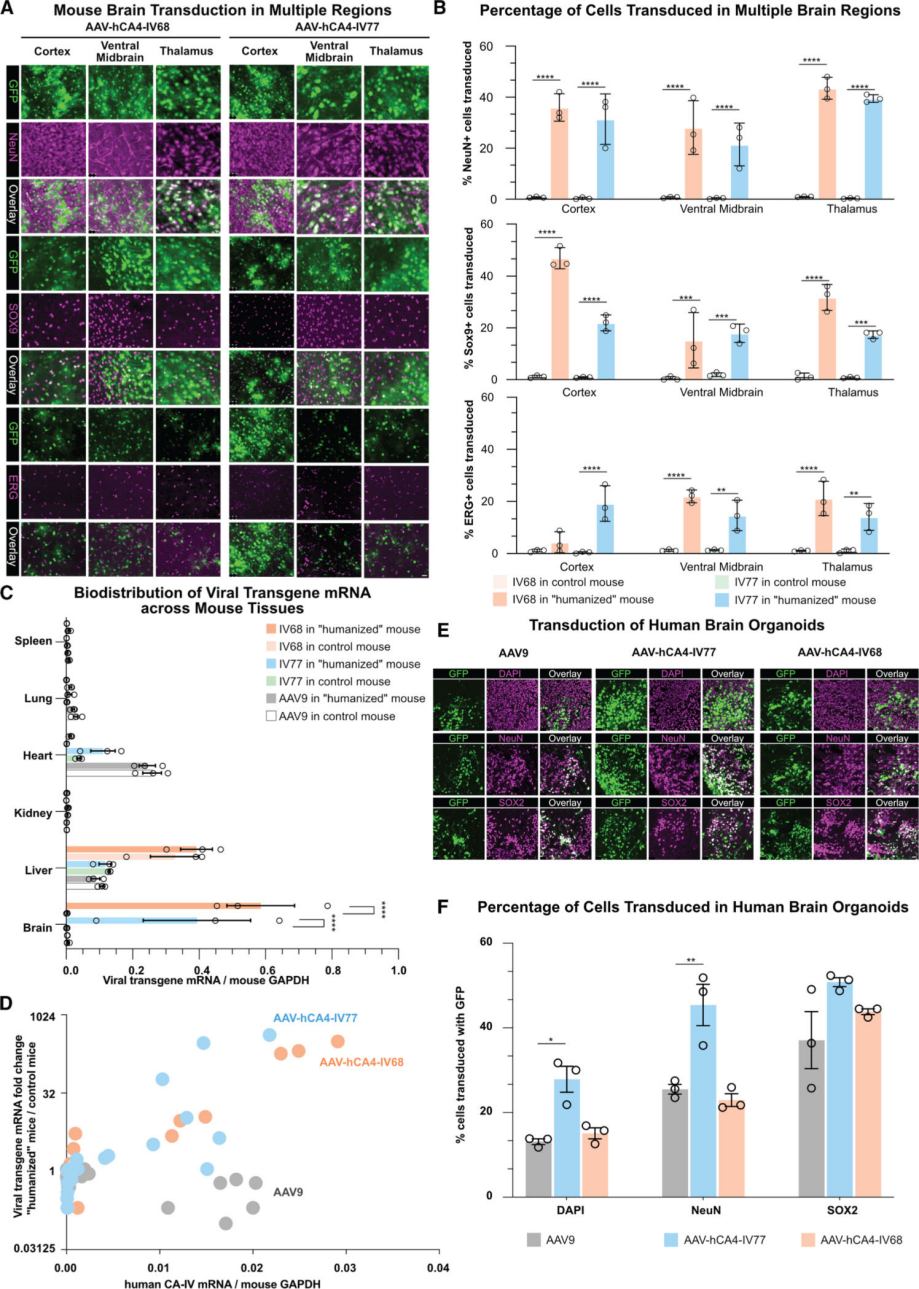
Validation in a “humanized” mouse model (A) Representative images showing transduction across multiple brain regions (cortex, ventral midbrain, and thalamus) for AAV-hCA4-IV68, AAV-hCA4-IV77, and AAV9 in control and “humanized” mice. Green fluorescence (NLS-EGFP) indicates transduced cells, NeuN marks neurons, SOX9 marks astrocytes, and ERG marks endothelial cells. Scale bars, 20 μm. (B) Quantification of transduction efficiency across brain regions. The top panel shows the percentage of NeuN+ neurons transduced, the middle panel shows the percentage of SOX9+ astrocytes transduced, and the bottom panel shows the percentage of ERG+ endothelial cells transduced for each AAV variant in control and “humanized” mice. Data are represented as mean ± SEM. Statistical significance was determined using two-way ANOVA followed by post-hoc multiple comparison tests (Tukey’s test). Asterisks indicate levels of significance (**p < 0.01, ***p < 0.001, and ****p < 0.0001). All experiments were performed in biological triplicate. (C) Biodistribution of viral transgene mRNA across multiple organs (brain, liver, kidney, spleen, heart, and lung) in control and “humanized” mice. Data are presented as the ratio of viral transgene mRNA to mouse host genome *GAPDH* transcript. Data are represented as mean ± SEM. Statistical significance was determined using two-way ANOVA followed by post-hoc multiple comparison tests (Tukey’s test). Asterisks indicate levels of significance (****p < 0.0001). All experiments were performed in biological triplicate. (D) Correlation between human CA-IV expression level and viral genome distribution across organs. The fold change in viral transgene mRNA levels between “humanized” and control mice is plotted against human CA-IV transcript copies. AAV-hCA4-IV77 (blue circles) and AAV-hCA4-IV68 (orange circles) show a positive trend with increased transcript levels in organs expressing higher amounts of CA-IV, while AAV9 (gray circles) shows no correlation with CA-IV expression. (E) Representative images of human brain organoid transduction comparing AAV9, AAV-hCA4-IV77, and AAV-hCA4-IV68. Organoids were exposed to AAVs in culture media for 2 weeks. Green fluorescence (EGFP) indicates transduced cells, DAPI marks cell nuclei, NeuN marks mature neurons, SOX2 marks neural progenitors, and the overlay shows the co-localization. Scale bars, 20 μm. (F) Quantification of transduction efficiency in human brain organoids. Bar graphs show the percentage of total cells (DAPI+), mature neurons (NeuN+), and neural progenitors (SOX2+) transduced by AAV9, AAV-hCA4-IV77, and AAV-hCA4-IV68. Data are represented as mean ± SEM. Statistical significance was determined using unpaired *t* tests. Asterisks indicate levels of significance (*p < 0.05 and **p < 0.01). All experiments were performed in biological triplicate, with individual dots representing independent organoids.

We proceeded with individual validation of IV68 and IV77 in our “humanized” mouse model (Figure 3D). We intravenously administered 5 × 10^11^ vg of AAVs carrying EGFP with a nuclear localization signal (NLS) into both control and “humanized” mice. Three weeks post-injection, we evaluated EGFP signal in brain tissue. The negative control AAV9 showed consistently poor brain transduction in both “humanized” and control mice (Figure 3D). In contrast, both IV68 and IV77 demonstrated significantly enhanced and widespread transduction throughout the brains of “humanized” mice compared to control mice. Unlike PHP.eB and IV68, small patches of particularly strongly transduced neurons and astrocytes were observed brain-wide after injection with IV77. Analysis across multiple brain regions, including cortex, ventral midbrain, and thalamus, revealed robust transduction by both variants, with neuronal coverage ranging from 20% to 40% and astrocytic coverage from 15% to 45% in human-CA-IV-expressing mice (Figures 4A and 4B). Notably, EGFP signal from both variants was minimal in control mouse brains, requiring higher laser intensity settings to detect their signal, which was similar to that of AAV9.

To further characterize these variants, we examined the biodistribution of IV77 and IV68 and their dependence on human CA-IV delivered by AAV-BR1, which retains AAV2’s broad peripheral tropism. We quantified viral transgene mRNA across multiple organs, including the brain, liver, kidney, spleen, heart, and lung (Figures 4C, S5A, and S5B). As expected, AAV9 cargo transcript levels showed no significant difference between control and “humanized” mice, with predominant expression in the liver and heart and relatively low levels in the brain. In contrast, IV77 and IV68 demonstrated a clear brain-enriched pattern, reaching levels equivalent to 40% and 60% of the mouse host genome *GAPDH* transcript in “humanized” mice, respectively, while showing minimal expression in control mice. In the brain, both IV77 and IV68 showed over 100-fold higher transgene mRNA levels in “humanized” mice compared to AAV9, and more than 250-fold higher expression compared to levels in control mice (Figures S5A and S5B). Additionally, IV68 exhibited slightly increased liver transduction compared to both IV77 and AAV9.

Furthermore, IV77 and IV68 exhibited comparable transcript levels in the liver, spleen, lung, and kidney between “humanized” and control mice, with moderately elevated expression in the heart of “humanized” mice. The negligible brain transduction of IV77 and IV68 in control mice also suggests that our engineered AAVs specifically recognize human CA-IV but not mouse CA-IV. This species specificity highlights a key advantage of our selection pipeline—the ability to engineer variants optimized specifically for the intended target species.

To further understand the relationship between human CA-IV expression and transduction efficiency, we investigated whether the transduction patterns of IV77 and IV68 in various organs correlated with human CA-IV expression levels. We plotted the fold change in viral transgene mRNA levels against human CA-IV transcript copies across the brain, liver, kidney, spleen, heart, and lung tissues (Figure 4D). While AAV9 showed no correlation with CA-IV expression, IV77 and IV68 demonstrated a positive trend, with increased transcript levels in organs expressing higher amounts of CA-IV. This analysis could provide insights into the potential biodistribution profile of IV77 in human applications, since human CA-IV expression data are available.^37,55^

As only the BBB was “humanized” in our mouse model, we investigated IV77 and IV68’s potential performance on human brain cells after BBB transcytosis using human brain organoids.^56–58^ Human embryonic stem cells were differentiated first into cortical neuron precursors, then aggregated and matured into 3D organoids. AAV variants packaging EGFP under the control of a CAG promoter were added to the culture media (not injected into the organoids) and imaged after 2 weeks of incubation.

Compared to AAV9, which showed moderate transduction in the organoid, AAV-hCA4-IV77 showed about 2-fold greater overall cell transduction, with a similar improvement in mature neurons (NeuN+) (Figures 4E and 4F), while IV68 performed similarly to AAV9. Since the human CA-IV level in human brain neurons is minimal (Figure S1A), the improved transduction of IV77 may arise from a modulation of the capsid’s naturally evolved receptors.^48^

## DISCUSSION

For the past decade, *in vivo* directed evolution has been the primary method for developing AAV variants.^17,18^ This approach, which can identify capsid variants with enhanced properties without knowledge of the underlying mechanisms, has led to significant advancements in AAV technology. However, it faces challenges in translation due to genetic context differences between species. Unless directed evolution is to be performed in humans, there is a significant limitation in the clinical application of engineered AAV capsids identified in this manner. One strategy to minimize this risk is to select AAV variants in multiple species, aiming to identify vectors that utilize BBB crossing mechanisms that are highly conserved; however, these may not be the most efficient in a given species.

Here, we utilize a more recent approach to address this challenge, engineering optimal AAVs for the intended species by engaging a known BBB transcytosis receptor.^31,43^ The choice of luminal BBB receptor is critical to vector potency and safety. Directed evolution of AAVs was greeted with enthusiasm in large part because it offered an alternative to the handful of transcytosis receptors such as TfR1 and INSR that had been known for decades.^54,59–61^ As our understanding of AAV transcytosis mechanisms and BBB biology advances,^32–35,37,38,55,62–68^ opportunities for targeted, mechanism-based engineering strategies are emerging. The approach we describe here brings mechanism-guided AAV engineering methods, similar to those recently applied for targeting TfR1,^43^ to a promising recently identified transcytosis receptor utilized by some of the most potent mouse vectors: CA-IV.^18^

Crucially, CA-IV’s expression pattern offers unique benefits over TfR1. Unlike TfR1, which is also expressed in non-brain tissues and non-endothelial brain cells, CA-IV is predominantly restricted to brain endothelial cells and spans brain regions (Figures S1A and S1B).^37,38^ This expression pattern has the potential not only to enhance BBB targeting but also to support future engineering of vectors specific to cell types within the brain. This potential for specificity is supported by the transduction patterns of human CA-IV-targeting AAVs in our “humanized” mouse model, where vectors’ peripheral transduction tracks human CA-IV expression, thus providing valuable predictive information for potential biodistribution profiles in human translational applications.

Interestingly, the most effective BBB-crossing human CA-IV-dependent variants that we identified (AAV-hCA4-IV68 and AAV-hCA4-IV77) arose from a hybrid *in vitro/in vivo* selection pipeline, rather than a fully *in vitro* approach. While several top-ranked variants from fully *in vitro* selection (Alpha 48, 49, and 62) showed strong human CA-IV-dependent boosts in cell transduction, they performed poorly *in vivo*. Conversely, the top two *in vivo* capsids were relatively lower ranked *in vitro* round 1 performers within the top 0.01% (10,000 variants) chosen for inclusion in round 2 selection. This highlights the value of *in vivo* selections using “humanized” mice and suggests a limitation for AAV engineering based solely on *in vitro* selection wherein stringent thresholds for *in vitro* performance may risk missing potent *in vivo* vectors. Nevertheless, *in vitro* pre-selection retains value, as it is fast and cost effective and enriches the library for vectors focused on the receptor of interest prior to *in vivo* screening, thereby increasing the likelihood of identifying receptor-dependent BBB crossers. Importantly, our findings do not imply that all top-ranked *in vitro* performers will perform poorly *in vivo*, and our results are consistent with recent work showing that not all highly enriched LY6A-binding AAV variant sequence clusters perform well *in vivo*.^31^ Due to practical constraints, we could not individually evaluate all top-performing AAVs from *in vitro* selection. Having tested only four variants (Alpha 41, 48, 49, and 62), it is possible that we missed candidates which would excel both *in vitro* and *in vivo* (as we indeed show for our controls PHP.eB and 9P31). Other therapeutic modalities, such as antibodies, have found that stronger receptor binding does not always positively correlate with more efficient BBB transcytosis.^54^ Prior work has also shown that engineering PHP.eB for even stronger LY6A binding can result in loss of brain potency.^69^ It is possible that our findings with IV77 and IV68 in relation to Alpha 48, 49, and 62 reveal a similar phenomenon with human CA-IV. It is also possible that the *in vitro* pull-down selection did not fully recapitulate key characteristics of *in vivo* CA-IV engagement. For example, a weak or transient CA-IV interaction on-column might become more effective through multivalent contacts on vascular endothelial membranes, or via stabilization by native CA-IV features (e.g., local pH, post-translational modifications) or cofactors (e.g., membrane or serum proteins) present *in vivo*. Such factors may also account for the lack of observable receptor binding by IV68 via SPR. Future work will be necessary to understand the precise mechanism of IV68’s enhanced brain potency.

The “humanized” mouse model used here offers a valuable bridge between traditional *in vitro* assays and direct human testing, providing a more physiologically relevant environment for human CA-IV. Notably, our approach of using endothelial cell-specific AAVs to deliver human receptors offers a substantially faster development timeline than traditional transgenics (weeks versus months to years) and greater flexibility to test emerging human receptors. This approach could serve as a template for developing other receptor-targeted therapies, potentially reducing late-stage clinical failures by better predicting human performance during preclinical development.

In summary, here we describe the development of AAV variants specifically engineered for human applications, with a focus on enhanced delivery to the brain by crossing the BBB via the recently identified receptor CA-IV. Integrating rapid *in vitro* engineering and “humanized” *in vivo* selections, we successfully identified human CA-IV-specific capsid variants capable of efficient transcytosis, with IV77 demonstrating a 100-fold increase in brain transduction efficiency compared to AAV9 in “humanized” mice and a 250-fold increase compared to control mice. Our work addresses the cross-species challenges commonly encountered with murine or NHP-selected AAV variants while highlighting the promise of emerging BBB transcytosis receptors identified via those products of directed evolution.

### Limitations of the study

Several limitations to the application of the vectors identified in this study remain. Most prominently, the potential impacts of differences in receptor expression levels in the BBB and peripheral tissues between our “humanized” mouse model and humans are unknown. This may be resolved in future work through the development of CA-IV-dependent vectors that could be characterized in wild-type primates. Our study also does not address the potential impacts of AAV binding on CA-IV enzymatic activity. The safety profile of FDA-approved oral carbonic anhydrase inhibitors for conditions such as altitude sickness, however, suggests that this should not preclude vectors that similarly inhibit the enzyme.

## RESOURCE AVAILABILITY

### Lead contact

Further information and requests for resources and reagents should be directed to and will be fulfilled by the lead contact, Viviana Gradinaru (viviana@caltech.edu).

### Materials availability

Key AAV capsid plasmids generated in this study have been deposited at Addgene (see key resources table for accession numbers). All other constructs and tools are available through the Beckman Institute CLOVER Center (clover.caltech.edu).

### Data and code availability

- Next-generation sequencing datasets generated in this study have been deposited at the Sequence Read Archive and are publicly available as of the date of publication. Accession numbers are listed in the key resources table.
- Code used for analyzing capsid selections and processing *in vitro* transduction images is available on GitHub and listed in the key resources table.
- Any additional information required to reanalyze the data reported in this paper is available from the lead contact upon request.

## STAR★METHODS

### EXPERIMENTAL MODEL AND STUDY PARTICIPANT DETAILS

#### Animals

All animal experiments were conducted with approval from the Caltech Animal Care and Use Committee and the California State Polytechnic University Pomona Animal Care and Use Committee, adhering to all applicable ethical guidelines. The mouse strains C57BL/6J (000664), DBA/2J (000671), CBA/J (000656) and non-obese diabetic (NOD)/ShiLtJ (001976) were obtained from The Jackson Laboratory (JAX). Male mice 5–8 weeks old were used. As such, the influence of sex on the results of the study is unresolved.

#### Cell lines

HEK293T cells were cultured in Dulbecco’s modified Eagle’s medium (DMEM) supplemented with 2 mM GlutaMAX, 5% fetal bovine serum and 1% nonessential amino acids (NEAAs) at 37°C in 5% CO_2_. AAV-MAX suspension cells were cultured in Viral Production Medium with 4 mM GlutaMAX at 37°C in 8% CO_2_. Expi293 cells were cultured in Expi293 Expression Medium at 37°C in 8% CO_2_. Human embryonic stem cells (hESCs) were cultured in E8 medium on Vitronectin-coated plates as described previously.^70^ Using the ‘dual-SMAD’ induction protocol, hESCs were differentiated into cortical neuron precursors in monolayer culture.^71^ The precursors were then dissociated into single cells and 100,000 precursors were placed into each well of V-bottom low-attachment plates to form cortical organoids. Afterward, the organoids were transferred to 10 cm dishes and placed on a shaker for long-term culture and maturation.^72^ Cell lines were neither authenticated nor tested for mycoplasma contamination.

### METHOD DETAILS

#### Plasmids

Sequences encoding AAV capsid variants were cloned into the pUCmini-iCAP plasmid (Addgene ID: 103005). Alpha 49, AAV-hCA4-IV77 and AAV-hCA4-IV68 have been deposited to Addgene (Addgene ID: 244910, 244907 and 244906). The open reading frames of the CA-IV and LY6A receptors were cloned into the pcDNA3.1 plasmid (ThermoFisher) for production in HEK293 mammalian cells. pAAV-CAG-hCA4-miR122 and pAAV-CAG-tdTomato-miR122 for making “humanized” mouse model are also deposited to Addgene (Addgene ID: 244908 and 244909). AAV capsid libraries were constructed using a library backbone containing CMV promoter, P41 promoter and TelN digestion sites, following previously published methods.^17,18^ These library plasmids can be obtained upon request from the CLOVER Center at the California Institute of Technology (Caltech).

#### AAV vector production

AAV packaging and purification were carried out as previously described, with modifications to utilize AAV-MAX suspension cells (ThermoFisher, A51217).^73^ In brief, recombinant AAV was produced by triple transfection of cells in suspension using the VirusGEN AAV Kit with RevIT enhancer (Mirus Bio, MIR 8007) at a molar ratio of transgene: AAV capsid: pHelper = 1:2:0.5, as per the manufacturer’s instructions. The total DNA amount was 2 μg per mL of cells. Virus-producing cells and medium were harvested 72 h post-transfection. Viral particles were purified using iodixanol step gradient columns followed by ultracentrifugation, as previously detailed.^73^ AAV-hCA4-IV77 required a final buffer formulation of DPBS with 0.001% (vol/vol) Pluronic F-68 and 300 mM NaCl for stable storage at 4°C. The purified AAVs were quantified through droplet digital PCR (ddPCR, Bio-Rad) following Addgene’s protocol with modifications. Briefly, AAV samples were serially diluted in nuclease-free water to achieve a suitable concentration for ddPCR analysis. The reaction mixture was prepared using 10 μL of ddPCR Supermix for Probes (Bio-Rad), 1 μL of primers binding to ITR regions (final concentration 900 nM), 5 μL of the diluted AAV sample, 10 μL 2X ddPCR EvaGreen Supermix (Bio-Rad) and nuclease-free water to a final volume of 20 μL. The mixture was then partitioned into droplets using the QX200 Droplet Generator (Bio-Rad). Thermal cycling was performed under the following conditions: 95°C for 10 min, followed by 40 cycles of 95°C for 30 s and 60°C for 1 min, and a final hold at 90°C for 5 min. After cycling, droplets were read using a QX200 Droplet Reader (Bio-Rad), and the concentration of positive droplets was analyzed using QuantaSoft software (Bio-Rad). The viral genome titer was calculated using the Poisson distribution, accounting for dilution factors and sample volumes.

#### BBB receptor single cell transcriptomic data analysis

**Data Acquisition and Processing.** Transcriptomic data were obtained from a previous publication,^55^ which profiled cellular diversity across the adult human brain using single-nucleus RNA sequencing. The dataset, including raw count matrices and metadata, was downloaded from CELLxGENE.^74^ All analyses were conducted using Scanpy,^75^ implemented in Python v3.12. **Cell Type Expression Analysis.** To investigate the expression of genes implicated in BBB transcytosis across brain cell types, raw count data were normalized using the normalize_total function in Scanpy and log transformed. Gene expression values were subsequently scaled across all cells prior to visualization. Predefined cell type annotations from the original study were used without modification. A dotplot was generated using Scanpy’s sc.pl.dotplot function to represent scaled expression levels of selected genes across major brain cell types. **Regional Endothelial Cell Analysis.** To examine the spatial distribution of CA-IV expression across brain regions, endothelial cells were subsetted from the dataset based on the cell type annotations provided by Siletti et al.^55^ Average CA4 expression was calculated within each brain region and then mapped onto a modified schematic^55^ using Adobe Illustrator, with expression levels represented as a continuous color gradient.

#### Pulldown-based AAV selection

GPI anchors in human and mouse CA-IV and mouse LY6A were identified using PredGPI software. These anchors were removed to enable secretion of the receptor proteins into the culture medium from AAV-MAX HEK293 cells (ThermoFisher, A51217). An HA tag was added to the C terminus to facilitate protein enrichment on HA resin. The receptor proteins with HA tags were expressed in AAV-MAX cells in suspension, with transfection carried out using the VirusGEN AAV Kit and RevIT enhancer as described above. The cell medium, containing the secreted receptors, was collected and stored at −80°C.

Pulldown-based selection was modified from a previously-published protocol.^76,77^ 5 μL of magnetic HA resin slurry (ThermoFisher) was incubated with the AAV library (2×10^10^ to 2 × 10^11^ vg) in 3.5 mL of medium containing the secreted receptors, or in fresh medium, and incubated overnight at 4°C with 0.1% Tween 20 on an end-to-end rotator. After incubation, the resin was pelleted and washed four times with TBS-T (20 mM Tris, pH 7.6, 150 mM NaCl, 0.05% Tween 20). Following the final wash, the resin was spun down to remove any remaining buffer, and 150 μL 1X DNA/RNA Shield (Zymo, R1100–50) was added. Capsids were lysed by heating at 95°C for 10 min. Proteinase K (5% v/v) was added after cooling, and samples were incubated at 25°C for 15 min to degrade capsid proteins. DNA was extracted using the Quick-DNA/RNA viral kit (Zymo, D7021) following the manufacturer’s protocol. The extracted DNA was then processed to add flow cell adaptors around the diversified peptide region using the following primers:

forward, ACACTCTTTCCCTACACGACGCTCTTCCGATCTACAAGTGGCCACAAACCACCA, reverse, GTGACTGGAGTTCAGAC GTGTGCTCTTCCGATCTCCTTGGTTTTGAACCCAACCGG.

Dual Index primers (NEB, NEBNext Multiplex Oligos for Illumina) were used to add indexes for next-generation sequencing (NGS). The samples were then sent to Novogene or the Caltech Millard and Muriel Jacobs Genetics and Genomics Laboratory for NGS analysis.

#### Cell transduction-based AAV selection

HEK293T adherent cells were transfected in 6-well plates with either receptor or scramble plasmids using Lipofectamine 3000 (ThermoFisher) according to the manufacturer’s protocol. Two days post-transfection, an AAV library (1 × 10^9^ to 1 × 10^10^ vg) was added to the cells. After 24 h, the cells were pelleted and washed with PBS to remove residual extracellular AAVs. RNA was extracted using the RNA Clean Kit (Zymo) following the manufacturer’s instructions. The extracted RNA was reverse transcribed into cDNA using Maxima H Minus cDNA Synthesis Master Mix (ThermoFisher, M1662). The cDNA was then prepared for NGS, as described above.

#### Cell transduction assay of AAV variants

HEK293T adherent cells were transfected in 6-well plates as described above. Two days post-transfection, cells were transferred to 96-well plates at 20% confluency. Simultaneously, AAV variants were added at concentrations of 5 × 10^8^ or 5 × 10^9^ vg per well. Cells were stained with NucBlue Live ReadyProbes Reagent (ThermoFisher, R37605) and imaged 24 h post-transduction using a Keyence BZ-X700 microscope. Fluorescence images were analyzed using an established quantification protocol to determine the percentage of transduced cells and the fluorescence intensity per transduced area.^32^

#### CA-IV protein expression and purification

CA-IV variants were cloned into a mammalian protein expression vector, with an Fc(human IgG1)-Myc-8xHis tag added to the C terminus of CA-IV. After sequence verification, the constructs were expressed using the AAV-MAX Production System (Gibco) according to the manufacturer’s instructions. At 96 h post-transfection, cells were pelleted, and the supernatant was filtered through a 0.5 μM filter. The supernatant was incubated with Ni-NTA agarose (Qiagen) for 2 h, after which the resin was washed with PBS containing 500 mM NaCl. The protein was eluted from the resin with PBS containing 150 mM imidazole.

#### Surface plasmon resonance

All SPR experiments were performed on a Sierra SPR-32 instrument (Bruker). Purified C-terminal Fc-fusions of CA-IV receptors were immobilized via the Fc-tag to a protein A-coupled sensor chip at a concentration of 100 nM in HBS-EP+ buffer (Teknova), reaching a capture level of 1000–1200 response units (RU). For each AAV construct, a 2-fold dilution series of AAV samples (starting at 1000 pM) was injected at a flow rate of 10 μL/min for 240 s, followed by a 600-s dissociation phase. A sensor chip regeneration step using 10 mM glycine at pH 1.5 was performed between each cycle. All kinetic measurements were analyzed with double reference subtraction. All experiments were performed in duplicate.

#### AAV Alpha 1 vector administration, tissue processing, and imaging

Adult C57BL/6J, DBA/2J, and NOD/ShiLtJ mice (*n* = 3), aged 6 to 8 weeks and of both sexes, received intravenous injections of 3×10^11^ vg AAV via the retro-orbital sinus. Mice were randomly assigned to groups receiving different AAVs. Following a 21-day incubation period, the animals were euthanized using CO_2_ narcosis and transcardially perfused, first with 10 mL of 0.1 M PBS (pH 7.4), followed by an equal volume of 4% PFA in the same buffer. Subsequently, the organs were extracted and subjected to overnight fixation in 4% PFA at 4°C. After fixation, the tissues were rinsed and preserved in 0.1 M PBS with 0.05% sodium azide, maintained at 4°C. Brain tissue was sectioned into 100 μm slices using a Leica VT100S vibrating blade microtome and imaged using a Nikon C2 confocal microscope and Nikon Elements Software at 20× magnification. Maximum intensity projections of 3 optical sections spaced 2 μm apart were used for quantification using ImageJ and Python.

#### AAV selection in “humanized” mouse model

Adult C57BL/6J mice (6–8 weeks old, male, *n* = 3) received intravenous injections of 1.5×10^12^ vg of AAV-BR1 carrying either CAG-tdTomato or CAG-human CA-IV constructs via the retro-orbital sinus. After four weeks, a second dose of 3×10^11^ vg of AAV pool of top candidates from *in vitro* selection was administered intravenously. Following a 3-week incubation period, mice were transcardially perfused with chilled PBS treated with 0.1% dimethyl pyrocarbonate (DMPC). Tissues were harvested, and AAV genomic RNA was extracted using TRIzol reagent and the Phasemaker System (ThermoFisher, A33251) according to the manufacturer’s protocol. The extracted RNA was reverse transcribed into cDNA using Maxima H Minus cDNA Synthesis Master Mix (ThermoFisher, M1662). The resulting cDNA was then prepared for NGS, as described above.

#### AAV characterization in “humanized” mouse model

To evaluate AAV tropism, we administered candidate AAV constructs individually (*n* = 3 per group per AAV variant). Four weeks following the delivery of tdTomato (control) or human CA-IV (“humanized”), mice were administered individual AAV constructs (RO, 5×10^11^ vg). Animals were subsequently perfused transcardially with chilled PBS treated with 0.1% dimethyl pyrocarbonate (DMPC). Harvested tissues were either post-fixed overnight in 4% PFA or snap-frozen at −80°C for parallel analyses. Fixed tissues were washed with PBS and sectioned at 100 μm with a vibrating blade microtome (Leica VT 1200S) for histological assessment. To assess cell-type tropism, sections were blocked for 1 h at room temperature (RT) with PBS containing 10% normal donkey serum and 0.2% Triton X-100. Tissues were then incubated overnight at 4°C with antibodies for neurons (Ms NeuN; Invitrogen, MA5–33103, 1:500), astrocytes (Rb SOX9; abcam, ab185966, 1:250), and endothelial cells (Rb ERG; Invitrogen, MA5–32036, 1:50). To assess effective delivery of human CA-IV, heat induced epitope retrieval was performed with Tris-based antigen unmasking solution (Vector Laboratories, H-3301–250) for 2 h at 95°C. Sections were then blocked and incubated with primary antibody, as described above, with anti-human CA-IV (ThermoFisher, 13931–1-AP, 1:500) and chicken anti-GFP (Aves Labs, GFP-1020, 1:500). Tissues were subsequently washed 3× with PBS-T and then incubated with fluorophore-conjugated secondary antibodies for two hours at RT. Nuclei were stained with DAPI (1:10,000) and tissues washed 3× with PBS-T and 3× with PBS before mounting with ProLong Diamond Antifade Mountant (Invitrogen, P36965). Slides were imaged using a spinning disk confocal microscope (Dragonfly, Andor; Fusion for software control) coupled with an sCMOS camera (Zyla, Andor). Images were acquired with ×10/×25 objectives (Leica) under the same conditions. Quantification of viral transduction was performed with the maximum intensity projection and analyzed using ImageJ and Python.

#### Biodistribution of AAV viral transgene mRNA and human CA-IV in mice

Frozen mouse tissues were homogenized and processed using TRIzol reagent and the Phasemaker System (ThermoFisher, A33251) for total RNA extraction. The extracted RNA was reverse transcribed into cDNA as described above. qPCR was performed to assess the biodistribution of viral transcripts using primers specific for EGFP (viral transgene), mouse GAPDH (host reference transcript), and human CA-IV (BR1-delivered transgene in “humanized” mice) with the following primers:

EGFP:

forward, CTATATCATGGCCGACAAGC, reverse, TGTTCTGCTGGTAGTGGTC.

GAPDH:

forward, AAATTCAACGGCACAGTCAA, reverse, CCCATTTGATGTTAGTGGGG.

Human CA-IV:

forward, AGGCACTGTCTAATATCCCC, reverse, GGAAGTAGTGCCTCAGTTTC.

#### Transduction of human cortical brain organoids

On day 70 of organoid culturing, 1 × 10^10^ vg of AAV variants encoding EGFP under control of a CAG promoter were added to the culture medium, with no direct injection into the organoids. Two weeks later, the organoids were cryo-sectioned (thickness 18 μm), fixed with 4% PFA and stained with primary antibody NeuN (Abcam, ab177487), SOX2 (ThermoFisher, 14–9811-82) followed by donkey anti-mouse 647 secondary antibody (Jackson ImmunoResearch, 715–606-151). Fluorescence images were captured using a spinning disk confocal microscope. Quantification was performed with ImageJ and Python.

### QUANTIFICATION AND STATISTICAL ANALYSIS

*In vitro* transduction (GFP intensity and % of GFP^+^ cells, Figures 2C, 2D, S4A, and S4B) was quantified with an established quantification pipeline.^32^ Comparisons between conditions used unpaired two-sample t-tests; significance was defined as *p* < 0.05 in both metrics. *In vivo* brain transduction (Figure 4B) was analyzed by two-way ANOVA, with Tukey’s post-hoc correction. Error bars indicate SD; biological replicates are noted in figure legends. qPCR-based biodistribution data (Figure 4C) were normalized to GAPDH, expressed as log fold changes, and assessed by two-way ANOVA with Tukey’s test. Analyses were performed in software Python and GraphPad Prism. Exact N, test details, and *p*-values appear in legends and method details.

## Supplementary Material

1

2

Supplemental information can be found online at https://doi.org/10.1016/j.celrep.2025.116419.

## Figures and Tables

**Table:** KEY RESOURCES TABLE

REAGENT or RESOURCE	SOURCE	IDENTIFIER

Antibodies		

NeuN	Invitrogen	Cat# MA5–33103; RRID: AB_2802653
SOX9	abcam	Cat# ab185966; RRID: AB_2728660
ERG	Invitrogen	Cat# MA5–32036; RRID: AB_2809330
anti-human CA-IV antibody	Thermo Fisher Scientific	Cat# 13931–1-AP; RRID: AB_2877990
anti-GFP antibody	Aves Labs	Cat# GFP-1020; RRID: AB_10000240
SOX2	Thermo Fisher Scientific	Cat# 14–9811-82; RRID; AB_11219471

Chemicals, peptides, and recombinant proteins		

Human CA-IV	This paper	N/A
Mouse CA-IV	This paper	N/A
Mouse LY6A	This paper	N/A

Critical commercial assays		

VirusGEN AAV Kit	Mirus Bio	Cat# MIR 8007
2X ddPCR EvaGreen Supermix	Bio-Rad	186–4034
Quick-DNA/RNA viral kit	Zymo Research	Cat# D7021
Dual Index primers	NEB	NEBNext Multiplex Oligos for Illumina
Maxima H Minus cDNA Synthesis Master Mix	Thermo Fisher Scientific	Cat# M1662
TRIzol reagent and the Phasemaker System	Thermo Fisher Scientific	A33251

Deposited data		

Next-generation sequencing datasets of AAV selection	This paper	Sequence Read Archive (Accession code: PRJNA1301436)

Experimental models: Cell lines		

AAV-MAX suspension cells	Thermo Fisher Scientific	Cat# A51217
HEK293T	ATCC	Cat# CRL-3216
Expi293	Thermo Fisher Scientific	A14635

Experimental models: Organisms/strains		

C57BL/6J	The Jackson Laboratory (JAX)	000664
CBA/J	The Jackson Laboratory (JAX)	000656
non-obese diabetic (NOD)/ShiLtJ	The Jackson Laboratory (JAX)	001976
human cortical brain organoids	This paper	N/A

Recombinant DNA		

pUCmini-iCAP-Alpha 49	This paper	Addgene: 244910
pUCmini-iCAP-hCA4-IV77	This paper	Addgene: 244907
pUCmini-iCAP-hCA4-IV68	This paper	Addgene: 244906
pAAV-CAG-hCA4-miR122	This paper	Addgene: 244908
pAAV-CAG-tdTomato-miR122	This paper	Addgene: 244909

Software and algorithms		

Prism 10.2.0	GraphPad	RRID:SCR_002798
Python	Python Software Foundation	RRID:SCR_008394; version 3.11
ImageJ (Fiji distribution)	FIJI	RRID:SCR_003070; version 2.9.0
PredGPI	Bologna Biocomputing Group	RRID:SCR_018363
QuantaSoft	Bio-Rad	RRID:SCR_025321, version 1.7.4
Nikon Elements Software	Nikon	RRID:SCR_014329, version 6.10.01
Code for analyzing capsid selections	This paper	GitHub (https://github.com/GradinaruLab/in-vitro-transduction-assay)
Code for processing *in vitro* transduction images	This paper	GitHub (https://github.com/GradinaruLab/in-vitro-transduction-assay)

Other		

magnetic HA resin	Thermo Fisher Scientific	Cat# 88837
1X DNA/RNA Shield	Zymo Research	Cat# R1100–50
Lipofectamine 3000	Thermo Fisher Scientific	Cat# L3000015
NucBlue Live ReadyProbes Reagent	Thermo Fisher Scientific	Cat# R37605
antigen unmasking solution	Vector Laboratories	Cat# H-3301–250
Dulbecco’s modified Eagle’s medium (DMEM)	Thermo Fisher Scientific	Cat# 10569044
GlutaMAX	Thermo Fisher Scientific	Cat# 35050061
Viral Production Medium	Thermo Fisher Scientific	Cat# A4817902
Expi293 Expression Medium	Thermo Fisher Scientific	Cat# A1435101

## Highlights

- Receptor-targeted AAV engineering and selection via hybrid *in vitro*/*in vivo* pipeline
- Human receptor expression in mice allows rapid assessment of translational AAVs
- Two AAVs targeting human CA-IV potently cross a humanized blood-brain barrier *in vivo*
