# Supplementary material for: AAVs targeting human carbonic anhydrase IV enhance gene delivery to the brain
Source: Cell Rep. Author manuscript; Available in PMC 2026 Feb 26. (PMC12942408; doi:10.1016/j.celrep.2025.116419)
Supplement: 1 [file NIHMS2125802-supplement-1.pdf]

Cell Reports, Volume *44*

Supplemental information

AAVs targeting human carbonic anhydrase IV enhance gene delivery to the brain

Changfan Lin, Xinhong Chen, Jonathan D. Hoang, Fiona Ristic, Yujie Fan, Seongmin Jang, Jin Hyung Alex Chung, Erin E. Sullivan, Tomasz Gawda, Bill Kavvathas, Irene Tran, Yitong Li, Andrew D. Steele, Timothy F. Shay, and Viviana Gradinaru

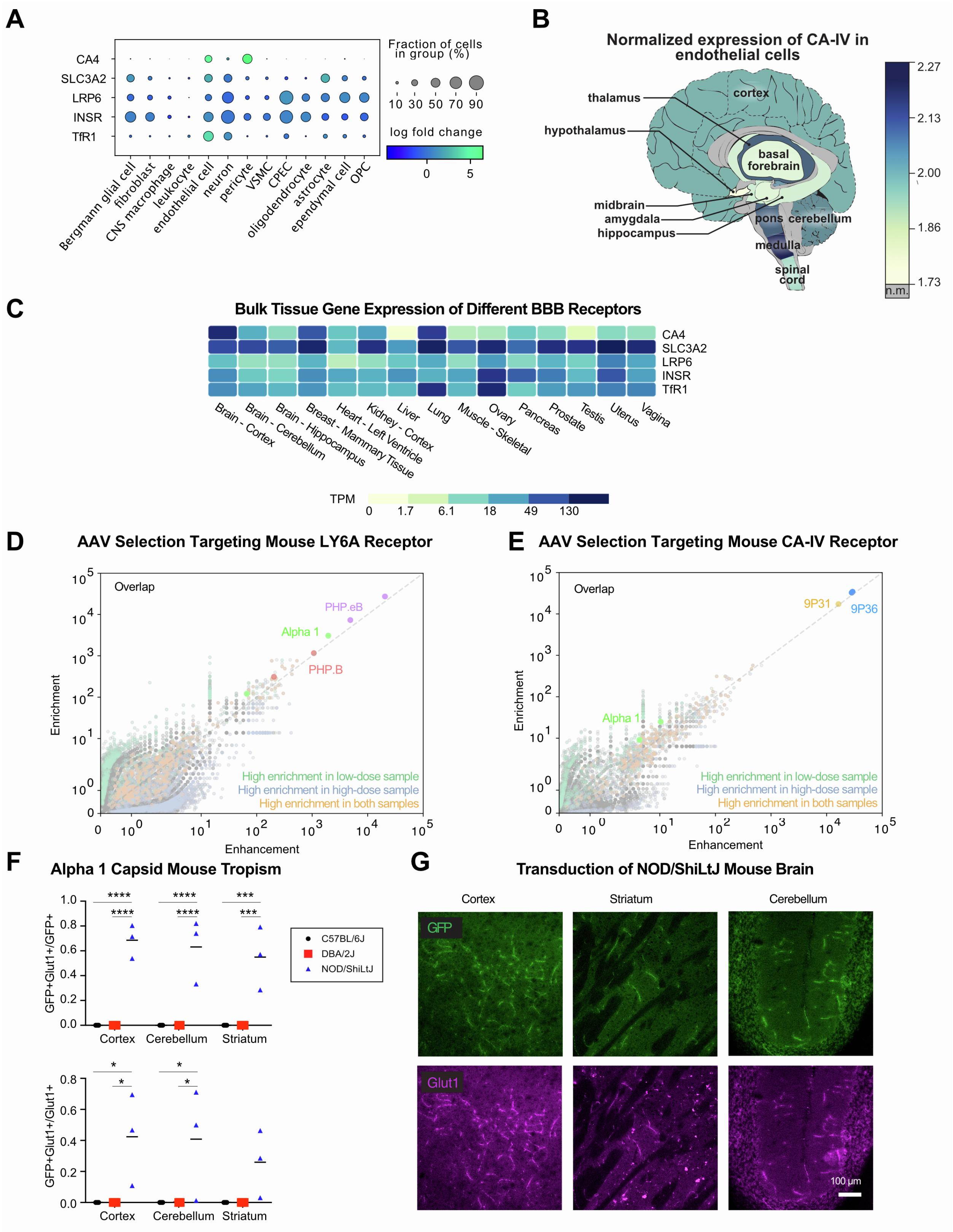

Figure S1: Blood-brain barrier receptor expression profiles and non-specific capsid variant characterization

(A) Comparative gene expression profiles of various blood-brain barrier receptors including carbonic anhydrase IV (CA4), solute carrier family 3 member 2 (SLC3A2), low-density lipoprotein receptor-related protein 6 (LRP6), insulin receptor (INSR), and transferrin receptor (TfR1) across different CNS cell types. VSMC, vascular smooth muscle cell. CPEC, choroid plexus epithelial cell. OPC, oligodendrocyte precursor cell. Dot size represents the fraction of cells expressing each receptor (10-90%), while color intensity indicates expression level (log fold change from 0 to 5). CA-IV shows a specific expression pattern across certain CNS cell types compared to other established BBB receptors.

(B) Heatmap showing the normalized expression of human CA-IV in endothelial cells across different regions of the human brain. CA-IV levels in gray colored regions were not measured (n.m.). Data modified from previous publication[52].

(C) Heatmap of gene expression levels for several BBB receptors, including CA-IV, solute carrier family 3 member 2 (SLC3A2), low-density lipoprotein receptor-related protein 6 (LRP6), insulin receptor (INSR), and transferrin receptor (TfR1), across various human tissues. Expression levels are quantified as Transcripts Per Million (TPM). Data from GTExPortal.

(D) Results of selection for AAV variants targeting the mouse LY6A receptor. Comparison of variant performance with low and high input doses reveals consistent enrichment of Alpha 1, PHP.B and PHP.eB.

(E) Results of selection for AAV variants targeting the mouse CA-IV receptor. Comparison of variant performance with low and high input doses shows reproducible enrichment of Alpha 1, 9P31 and 9P36.

(F) Quantification of GFP expression in different brain regions (cortex, cerebellum, and striatum) of three mouse strains (C57BL/6J, DBA/2J, and NOD/ShiLtJ) transduced with systemically-delivered Alpha 1 shows tropism inconsistent with known receptors. The top chart shows the percentage of transduced cells that are Glut1+ (endothelial cells), and the bottom the percentage of Glut1+ cells transduced. Animal number =3. Data are represented as mean +/- SEM. Statistical significance was determined using two-way ANOVA followed by post-hoc multiple comparison tests (Tukey's test). Asterisks indicate levels of significance (*$p<0.05$, ***$p<0.001$, ****$p<0.0001$).

(G) Representative fluorescence images of GFP expression (green) in the cortex, striatum, and cerebellum of NOD/ShiLtJ mice three weeks after systemic injection of 3×10^{11} vg/mouse of Alpha 1. Colocalization with Glut1 staining (magenta) highlights the transduction of endothelial cells.

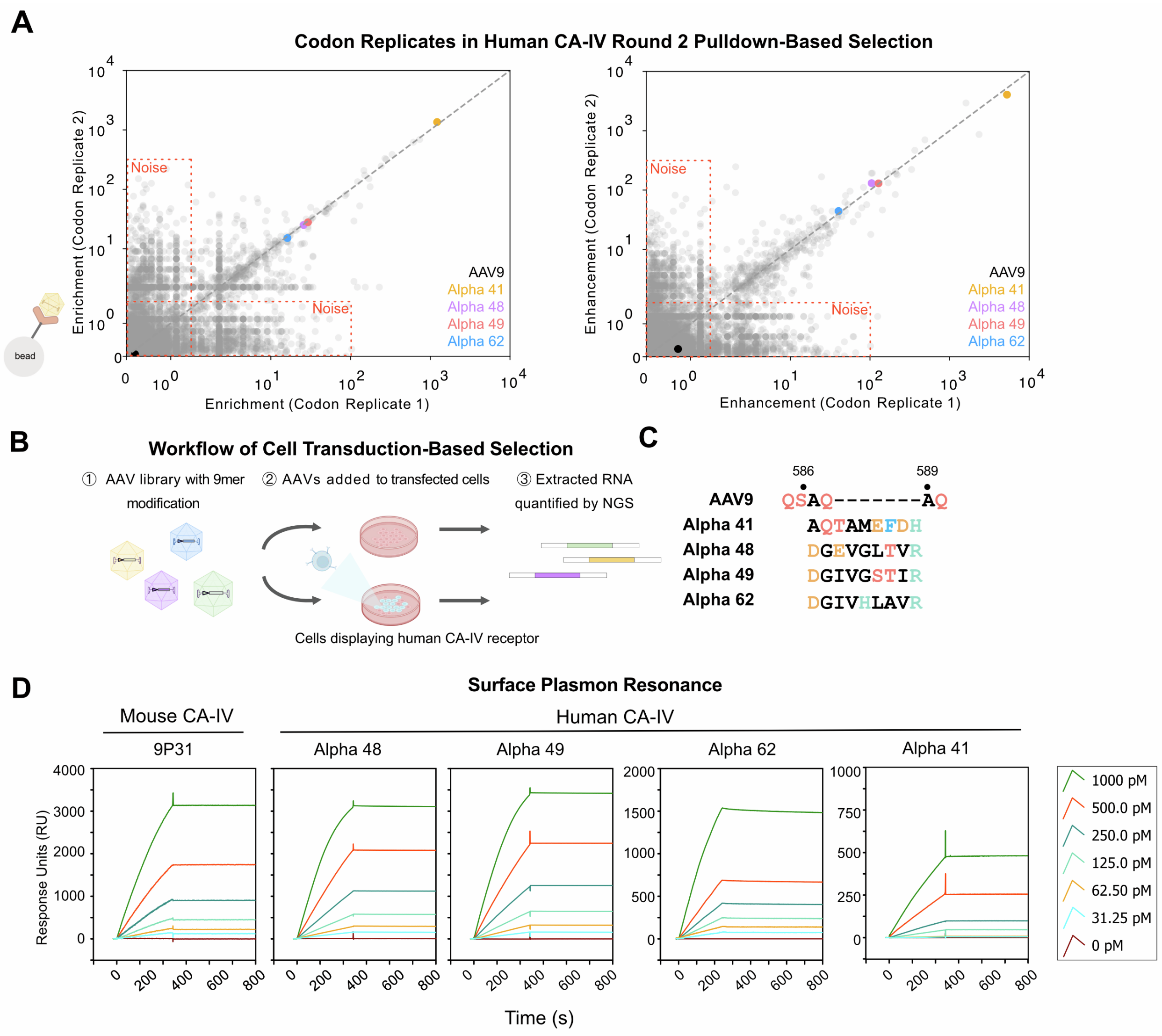

Figure S2: ***In vitro*** **selection and characterization of human CA-IV binders**

(A) Scatter plots showing the correlation between codon replicates of AAV variants after round 2 pulldown-based selection targeting human CA-IV. High correlation between replicates indicates reproducibility of selection. Variants Alpha 41, 48, 49, and 62 are highlighted.

(B) Schematic of cell transduction-based selection workflow for identifying AAV variants that effectively transduce cells expressing human CA-IV. (1) An AAV library with 9-residue modifications is prepared; (2) AAVs are added to cells transfected with either human CA-IV or a control plasmid; (3) RNA is extracted from transduced cells and quantified by next-generation sequencing (NGS) to identify variants showing receptor-dependent cell transduction.

(C) Amino acid sequences of the top AAV variants (Alpha 41, 48, 49, and 62) and reference AAV9 capsid showing the 9-residue modified regions between positions 586-589. Amino acids color-coded based on their properties: black, nonpolar aliphatic; blue, aromatic; red, polar uncharged; green, positively charged; orange, negatively charged.

(D) Surface plasmon resonance (SPR) binding profiles of AAV variants Alpha 41, 48, 49, and 62 and control (9P31) to mouse and human CA-IV receptors. Response units (RU) are plotted against time (s) at different concentrations (0-1000 pM) of each AAV variant.

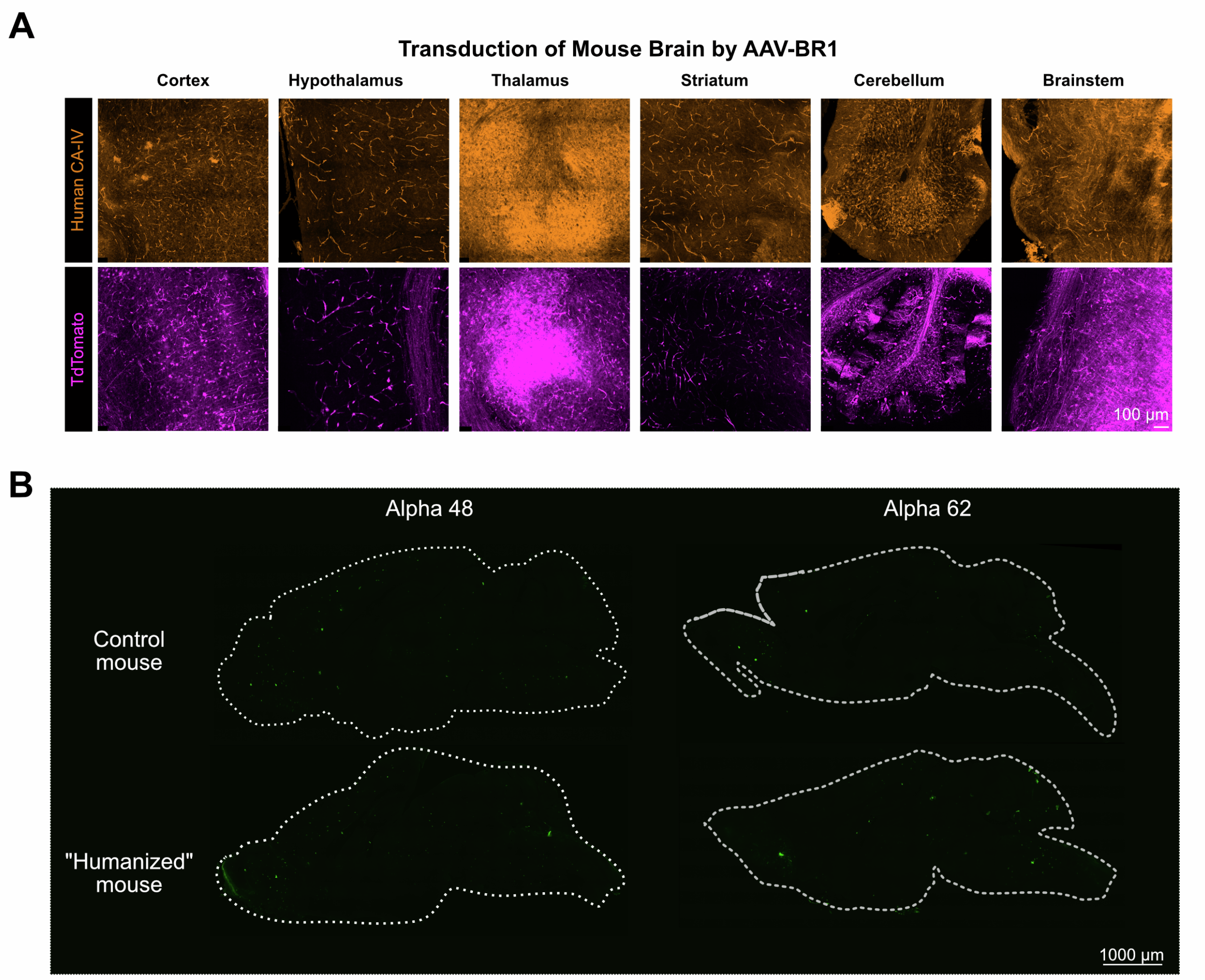

Figure S3: Validation of CA-IV expression in “humanized” mouse and characterization of CA-IV-binding AAVs

(A) Detection of protein expression for AAV-BR1 transgenes human CA-IV and TdTomato across multiple brain regions in the "humanized" mouse model. Brown staining indicates immunohistochemical detection of human CA-IV protein and purple staining indicates TdTomato direct fluorescence, which are distributed throughout cortex, hypothalamus, thalamus, striatum, cerebellum, and brainstem confirming successful expression of the human receptor in brain endothelial cells following AAV-BR1 delivery. Scale bar, 100 µm.

(B) Representative images of brain sections comparing Alpha 48 and Alpha 62 transduction in control mice versus "humanized" mice expressing human CA-IV. Brain outlines are indicated by dotted white lines. Scale bar, 1000 µm.

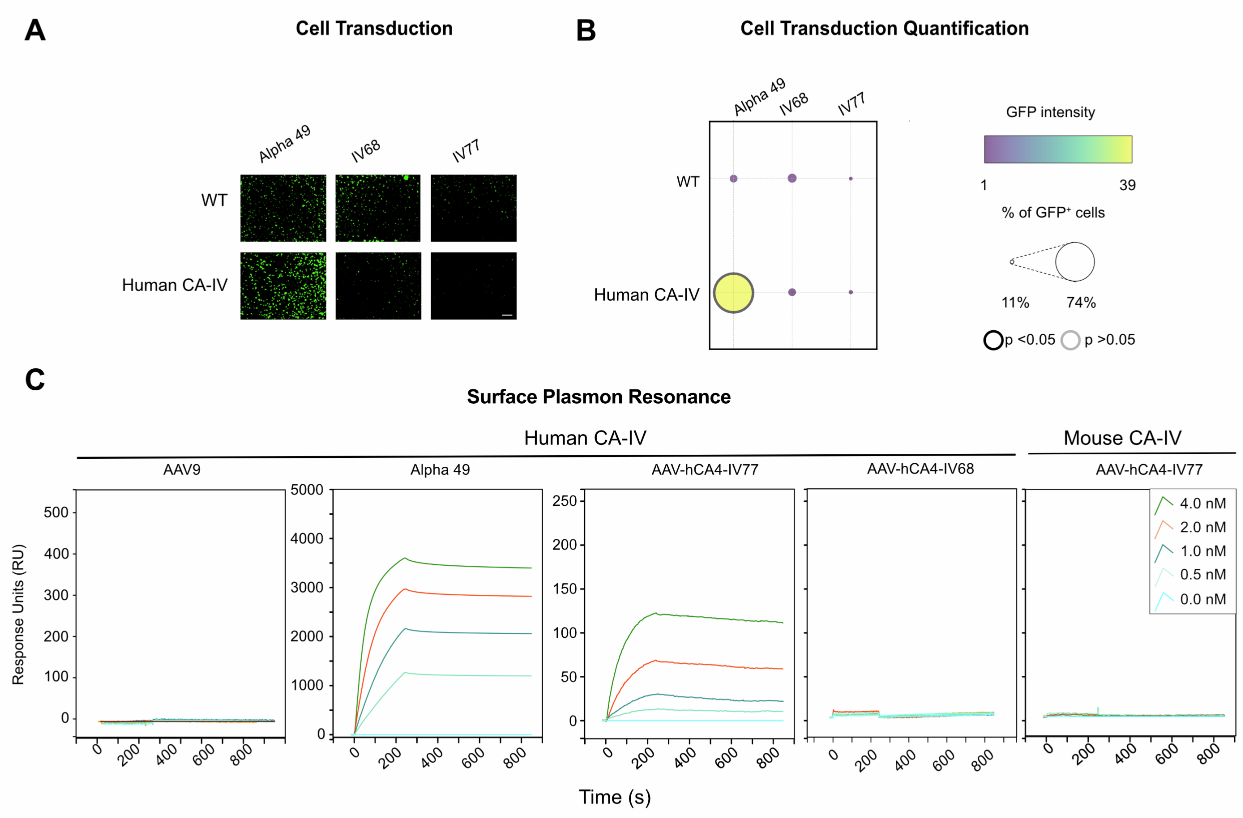

Figure S4: *In vitro* characterization of newly identified AAV variants

(A) Transduction of wild-type (WT) and human CA-IV-expressing HEK293 cells by Alpha 49, IV68 and IV77 carrying EGFP. Fluorescence images show significant enhancement of transduction by human CA-IV only for Alpha 49. Scale bar, 100 μm.

(B) Quantification of cell transduction (EGFP intensity and percentage of EGFP-positive cells), confirming enhanced transduction for Alpha 49. An independent sample t-test was performed between WT and human CA-IV expressing cells for each AAV variant. Dots with thick black borders represent a p-value of less than 0.05 in both EGFP intensity and the percentage of EGFP-positive cells. Dots with gray borders represent results that did not meet this significance threshold. Dot size represents percentage of EGFP-positive cells, with minimum and maximum values indicated in the legend. See Table S3 for quantification of transduction values.

(C) Surface plasmon resonance (SPR) binding profiles of AAV variants Alpha 49, AAV9, IV77 and IV68 to human or mouse CA-IV receptors. Response units (RU) are plotted against time (s) at different concentrations (0-4 nM) of each AAV variant.

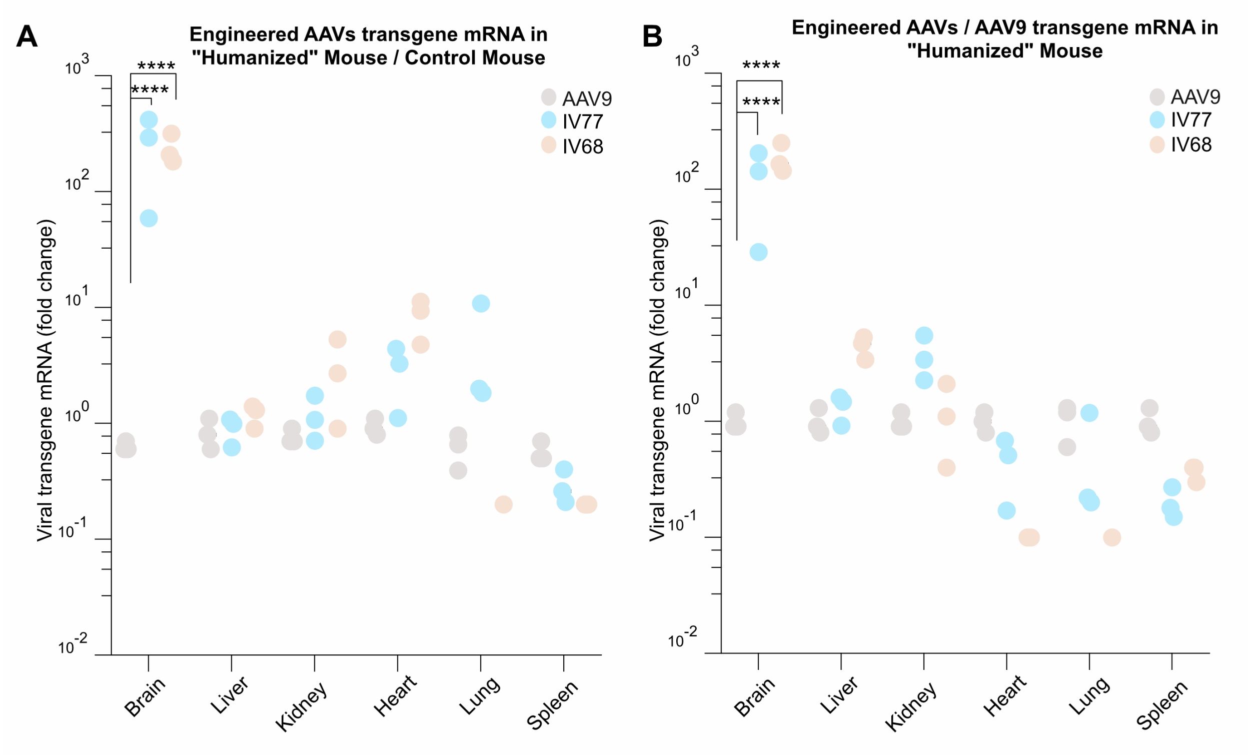

Figure S5: Fold change of AAV transgene mRNA in mouse model

(A) Comparison of AAV9, AAV-hCA4-IV77 and AAV-hCA4-IV68 viral transgene mRNA levels across multiple organs (brain, liver, kidney, heart, lung, and spleen) between "humanized" mice expressing human CA-IV and control mice. Data is presented as fold change on a logarithmic scale.

(B) Viral transgene mRNA levels of AAV-hCA4-IV77 and AAV-hCA4-IV68 compared to AAV9 in "humanized" mice expressing human CA-IV. AAV-hCA4-IV77 and AAV-hCA4-IV68 show significantly higher transcript levels in the brain (>100-fold compared to AAV9), with moderately elevated expression in heart, and comparable or lower levels in liver, kidney, lung, and spleen. Statistical significance was determined using two-way ANOVA followed by post-hoc multiple comparison tests (Tukey's test). Asterisks indicate levels of significance (****$p<0.0001$). All experiments were performed in biological triplicate.

Table S2: Individual replicate values for cell transduction assay in Figure 2D.

AAV variant	Cell Condition	GFP intensity replicate 1	GFP intensity replicate 2	GFP intensity replicate 3	% of GFP+ cells replicate 1	% of GFP+ cells replicate 2	% of GFP+ cells replicate 3
Alpha 41	WT	2567.0	2228.4	2222.3	0.11	0.11	0.12
Alpha 48	WT	3296.4	1486.1	2924.1	0.15	0.19	0.15
Alpha 49	WT	3875.8	4400.6	3026.5	0.16	0.16	0.16
Alpha 62	WT	4137.1	3661.9	3782.3	0.16	0.16	0.15
Alpha 41	Human CA-IV	2374.0	1457.2	1682.3	0.12	0.12	0.11
Alpha 48	Human CA-IV	53823.3	55449.6	48814.3	0.66	0.65	0.64
Alpha 49	Human CA-IV	70708.2	73050.7	69634.8	0.75	0.76	0.70
Alpha 62	Human CA-IV	24422.5	23909.5	25640.0	0.43	0.42	0.42

Table S3: Individual replicate values for cell transduction assay in Figure S4B.

AAV variant	Cell Condition	GFP intensity replicate 1	GFP intensity replicate 2	GFP intensity replicate 3	% of GFP+ cells replicate 1	% of GFP+ cells replicate 2	% of GFP+ cells replicate 3
Alpha 49	WT	10.38	4.29	5.05	0.009	0.007	0.010
IV68	WT	1.80	1.93	3.33	0.011	0.015	0.009
IV77	WT	1.91	1.16	1.06	0.003	0.003	0.002
Alpha 49	Human CA-IV	630.67	630.57	711.70	0.229	0.223	0.205
IV68	Human CA-IV	1.28	1.24	1.00	0.010	0.007	0.006
IV77	Human CA-IV	1.11	1.14	1.12	0.002	0.002	0.002
